# Is sling immobilization necessary after open Latarjet surgery for anterior shoulder instability? A randomized control trial

**DOI:** 10.1186/s13063-023-07180-9

**Published:** 2023-02-27

**Authors:** Patrick Goetti, Tiago Martinho, Antoine Seurot, Hugo Bothorel, Alexandre Lädermann

**Affiliations:** 1 Department of Orthopedic Surgery and Traumatology, Lausanne University Hospital and University of Lausanne, Avenue Pierre Decker 4, 1005 Lausanne, Switzerland; 2 Division of Orthopaedics and Trauma Surgery, La Tour Hospital, Rue J.-D. Maillard 3, CH-1217 Meyrin, Switzerland; 3 Department of Physiotherapy, La Tour Hospital, 1217 Meyrin, Switzerland; 4 Research Department, La Tour Hospital, 1217 Meyrin, Switzerland; 5 Faculty of Medicine, University of Geneva, Rue Michel-Servet 1, 1211 Geneva 4, Switzerland; 6 Division of Orthopaedics and Trauma Surgery, Department of Surgery, Geneva University Hospitals, Rue Gabrielle-Perret-Gentil 4, 1211 Geneva 14, Switzerland

**Keywords:** Rehabilitation, Physiotherapy, Recovery, Glenohumeral, PROMs, Results, Complications, Range of motion, Bony fusion, Graft

## Abstract

**Background:**

There is a current lack of knowledge regarding optimal rehabilitation and duration of sling immobilization after an open Latarjet procedure. A shift towards immediate self-rehabilitation protocols in shoulder surgery is observed to avoid postoperative stiffness and fasten return to sport. Avoiding sling immobilization could further simplify rehabilitation and provide an even faster return to activities of daily living and enhance patient satisfaction.

**Methods:**

This study is a single-center, randomized control trial. Sixty-eight patients will be instructed with the same standardized immediate postoperative self-rehabilitation protocol. Patients will be allocated 1:1 between a sling immobilization group for the first three postoperative weeks and no sling group without postoperative immobilization. The primary endpoint will be functional outcome at 6 months postoperative evaluated by the disease-specific Rowe score. Secondary endpoints will include baseline, 1.5-, 6-, and 12-month single assessment numeric evaluation (SANE) of instability score and visual analog pain scale (VAS). At the 6-month time point, graft bony union and position will be assessed by computed tomography. Motion capture technology will evaluate the baseline and 6-month postoperative range of motion. Finally, time to return to work and sport during the first postoperative year, along with patient satisfaction at one postoperative year, will also be recorded.

**Discussion:**

This study will allow further insights into the optimal rehabilitation protocol after open Latarjet surgery and enhance patient care by helping identify rehabilitation and coracoid graft-related factors influencing functional outcomes, bony union, range of motion, and patient satisfaction.

**Trial registration:**

The protocol was approved by the ethical committee board (CCER 2019–02,469) in April 2020 and by ClinicalTrials.gov (Identifier: NCT04479397) in July 2020.

## Administrative information

**Table:** 

Title {1}	**Is sling immobilization necessary after open Latarjet surgery for anterior shoulder instability? A randomized control trial**
Trial registration {2a and 2b}	The protocol was approved by the ethical committee board (CCER 2019–02,469) in April 2020 and by ClinicalTrials.gov (Identifier: NCT04479397) in July 2020
Protocol version {3}	Version 1, 26.06.2022
Funding {4}	FORE (Foundation for Research and Teaching in Orthopedics, Sports Medicine, Trauma and Imaging in the Musculoskeletal System). Grant#2022–29
Author details {5a}	Goetti Patrick^1^ MD, Martinho Tiago,^2^ MD, Antoine Seurot,^3^ PT, Bothorel Hugo,^4^ MEng, Lädermann Alexandre, MD.^2,5,6^ 1. Department of Orthopedic Surgery and Traumatology, Lausanne University Hospital and University of Lausanne, Avenue Pierre Decker 4, 1005 Lausanne, Switzerland 2.Division of Orthopaedics and Trauma Surgery, La Tour Hospital, Rue J.-D. Maillard 3, 1217 Meyrin, Switzerland 3. Department of Physiotherapy, La Tour Hospital, 1217 Meyrin, Switzerland 4. Research Department, La Tour Hospital, 1217 Meyrin, Switzerland 5. Faculty of Medicine, University of Geneva, Rue Michel-Servet 1, 1211 Geneva 4, Switzerland 6. Division of Orthopaedics and Trauma Surgery, Department of Surgery, Geneva University Hospitals, Rue Gabrielle-Perret-Gentil 4, 1211 Geneva 14, Switzerland
Name and contact information for the trial sponsor {5b}	Corresponding author Alexandre Lädermann, PD-MD Division of Orthopaedics and Trauma Surgery, La Tour Hospital, Av. J.-D. Maillard 3, CH-1217 Meyrin, Switzerland Telephone number: + 41 22 719 75 55 Fax number: + 41 22 719 60 77 E-mail address: alexandre.laedermann@gmail.com
Role of sponsor {5c}	Study design, randomization, data collection

## Introduction

### Background and rationale {6a}

Recurrent traumatic anterior shoulder instability occurs most commonly in young to middle-aged male athletes [1–5]. Bankart repair is a commonly performed surgical procedure but is associated with a high proportion of instability recurrence in the presence of glenoid and humeral bone loss [6–8]. On the other hand, the open Latarjet procedure is associated with low recurrence rates [9–13], notably thanks to biomechanical benefits which rely on a triple–stabilizing effect [14]. First, it acts as a bone graft procedure using the coracoid. Second, a sling effect is provided by the conjoint tendon passing through the subscapularis. Finally, additional glenohumeral stability can be restored at end range of motion by a capsulolabral reconstruction [15]. The Latarjet procedure was further reported to enable early return to sport compared to capsulolabral repair [16, 17]. However, the downsides are potential fracture and malunion of the coracoid bone graft along with reported stiffness secondary to the subscapularis split [18–21], and lower postoperative external rotation [16].

Recent research has highlighted the negative effect of immobilization on shoulder rehabilitation [16, 22, 23]. However, only few studies evaluated different rehabilitation programs after open Latarjet and their potential impact on complication rates, stiffness, and time to return to sport [8, 24–28]. Immobilization periods ranged from 0 to 3 weeks; different mobilization protocols were used (with and without supervision), but an early passive motion is suggested to avoid stiffness without increasing complication rates [29].

### Objectives {7}

This study will aim to compare immediate self-rehabilitation protocol using a sling to immediate self-rehabilitation without a sling after a Latarjet procedure for recurrent anterior shoulder instability. The effect of sling immobilization after open Latarjet surgery on self-reported functional outcomes, bone graft healing, range of motion, patient satisfaction, and time to return to sport and work will be analyzed. The hypothesis was that immediate self-rehabilitation without immobilization would result in improved functional outcome scores at 6 months follow-up compared to patients wearing a sling for the first three postoperative weeks. To our best knowledge, no study has sought to compare the usefulness of sling wearing after Latarjet procedure. Avoiding the sling could simplify rehabilitation and should provide a return to normal function faster, with greater satisfaction.

### Trial design {8}

This superiority prospective case–control clinical trial is randomized 1:1 between the sling-wearing and no sling groups.

## Methods: participants, interventions, and outcomes

### Study setting {9}

This study will be monocentric and performed at the department of orthopedic surgery of the Hôpital de la Tour in Meyrin, Geneva, Switzerland. The design is a two-arm, parallel group (sling versus no sling), randomized superiority trial with a 1:1 allocation ratio with as primary outcome 6-month functional outcomes as assessed by the disease-specific Rowe score.

### Eligibility criteria {10}

Participants fulfilling all of the following inclusion criteria are eligible for the study:

- Anterior shoulder instability with one or more of the following criteria:
  A glenoid bone defect > 20%
  Contact athlete
  Failed Bankart repair—either open or arthroscopic
- Informed consent as documented by signature,
- Age between 18 and 65 years.

The presence of any one of the following exclusion criteria will lead to the exclusion of the participant:

- Subscapularis tear,
- Polytrauma inducing significant limitation of a rehabilitation program,
- Significant other trauma of the involved upper member (e.g., associated scapular or clavicular fractures, acromioclavicular dislocation),
- Preoperative stiffness (defined by active and passive limitation in at least two directions, abduction and anterior elevation < 100°, external rotation < 20°, internal rotation < L3),
- Dislocation arthropathy,
- Patients suffering from symptomatic anemia or patients with severe cardiorespiratory insufficiency,
- Known or suspected non-compliance, drug or alcohol abuse,
- Patients incapable of judgment or under tutelage,
- Inability to follow the procedures of the study, e.g., due to language problems, psychological disorders, dementia, and contraindication for CT scan (i.e., pregnancy) of the participant,
- Enrolment of the investigator, their family members, employees, and other dependent persons.

### Who will take informed consent? {26a}

Informed consent will be collected by the scientific secretary (Anne-Sophie Guillarme, fondation.fore@latour.ch) at La Tour Hospital. The principal investigator (AL) will explain to each participant the nature of the study, its purpose, the procedures involved, the expected duration, the potential risks and benefits and any discomfort it may entail. Each participant will be informed that the participation in the study is voluntary and that he/she may withdraw from the study at any time and that withdrawal of consent will not affect his/her subsequent medical assistance and treatment. All participants for the study will be provided a participant information sheet and a consent form describing the study and providing sufficient information for participant to make an informed decision about their participation in the study. Patients will have until the surgery’s day to decide whether they will participate or not. The patient information sheet and the consent form will be submitted to the CEC and to the competent authority (as applicable) to be reviewed and approved. The formal consent of a participant, using the approved consent form, must be obtained before the participant is submitted to any study procedure. The participant should read and consider the statement before signing and dating the informed consent form, and should be given a copy of the signed document. The consent form must also be signed and dated by the investigator (or his designee, the scientific secretary) and it will be retained as part of the study records.

### Additional consent provisions for collection and use of participant data and biological specimens {26b}

No biological specimens are collected in this study, and no additional consent provision for collection and future use of participant data is included in the informed consent form.

## Interventions

### Explanation for the choice of comparators {6b}

The control group will wear a sling during the first three postoperative weeks. The interventional group will not wear any brace. We therefore compare the two extremes of reported immobilization periods currently reported in the literature for open Latarjet [8, 24–29]. Both groups will move passively during the first three postoperative weeks according to the Liotard self-rehabilitation protocol [24]. Investigators will perform clinical follow-ups at 1.5, 6, and 12 months. This period is considered sufficient to identify differences between both groups as they are expected to appear early in the postoperative phase [24]. Furthermore, reported graft union rate at 6 months is > 90%, and hardware-related complications are known to be detected early [8, 30].

### Intervention description {11a}

#### Surgical intervention

The surgeon (AL) and surgical techniques will be identical for all patients. The standardized surgical procedure uses a 90° angulated saw and an osteotome to harvest the coracoid [31]. A 1.5-cm stump of the coracoacromial ligament attachment was preserved onto the coracoid. Subscapularis was split at the junction between the upper two-thirds and lower one-third using scissors. Vertical capsulotomy was performed sharply. Whenever possible, the anterior labrum will be released inferiorly and tagged with sutures for subsequent repair. Two partially 1 cm apart threaded 4.5-mm cancellous screws (Arthrex, Naples, Florida, US) were used to fix the coracoid onto the anterior glenoid rim in 3–5 o’clock position using a free-hand technique. Lastly, capsulolabral repair and imbrication onto the coracoacromial ligament will be performed with the arm in external rotation with a posterior lever push to allow adequate tensioning [31].

#### Postoperative rehabilitation

In the sling group, patients will be instructed to wear the sling with the arm at the side of the body for 3 weeks. In the no sling group, patients will not wear any sling at all after surgery. Both groups will start immediate postoperative auto-mobilization in all axes during the first 3 weeks as described by Roulet et al. (Liotard’s protocol) [24]. The surgeon and physiotherapist will instruct the no sling group patients to not actively elevate and abduct their operated shoulder with only passive-assisted mobilization allowed. After 3 weeks, both groups will progress towards active mobilization, including external rotation of the shoulder, enabling a return to daily activities with the elbow at the side, and avoiding weightlifting. Sport-specific and strengthening exercises, including pulley, strings, and weights, will be allowed from the sixth postoperative week.

### Criteria for discontinuing or modifying allocated interventions {11b}

All adverse events must be transmitted to the sponsor-investigator (AL). He will manage or supervise reporting of adverse events and ensure that the follow-up of concerned patients is performed. He will be advised of all patients who withdraw or discontinue. He will plan additional monitoring visits, rehabilitation, or data collection if he judges it necessary. Trial data of the patient will be stored in a coded manner. The names of the patients will not be disclosed on CRF. A sequential unique patient number (UPN) will be attributed to each patient randomized into the trial. Identification of patients will be stored on a randomization list. Patients must be informed of and agree to data and material handling in accordance with Swiss data protection law.

### Strategies to improve adherence to interventions {11c}

In both groups, rehabilitation will be done at home by patients themselves according to Liotard protocol [24]. Patient compliance will be evaluated at each follow-up visit, confirming strict adherence to recommendations. For the sake of simplicity and study costs, no sensor will be used to evaluate the compliance with sling wearing.

### Relevant concomitant care permitted or prohibited during the trial {11d}

Concomitant care such as physiotherapy will be prohibited during the trial as it would add potential bias.

### Provisions for post-trial care {30}

There is no anticipated harm and compensation for trial participation. All patients in our trial will undergo shoulder surgery; they will all benefit from long-term follow-up if clinically requested by the operating physician and principal investigator (AL) and therefore receive adequate post-trial care.

### Outcomes {12}

As a primary outcome, we will evaluate functional outcomes using the disease-specific Rowe score [32]. The Rowe score consists of 100 points, of which 50 points are dedicated to evaluating stability, 20 points for mobility, and 30 points for the function. Functional outcomes will further be assessed using subjective scores in the form of self-administered questionnaires.

Secondary outcomes include pain on a visual analog scale (VAS) which is a widely used single-item test where a patient rates pain intensity between 0 and 10. The single assessment numeric evaluation (SANE) for instability [33], which is helpful for patient preoperative and postoperative monitoring, has also been correlated with patient pain, anxiety, and apprehension [34–37]. Patient satisfaction (are you satisfied, yes/no) will be assessed as well.

Secondary outcomes also include radiological criteria evaluated on X-rays and computed tomography (CT) at 6 months for bony union. The bony union will be assessed using the classification proposed by Hovelius et al. differentiating between union (absence of a radiolucent line), fibrous union (radiolucent line of 5 mm or less), and non-union (radiolucent line of more than 5 mm) [38]. Quantitative bone graft union will further be assessed using the CT-specific evaluation proposed by Samim et al. [39].

Bone graft characteristics, including lateral and medial overhang and graft height, will be assessed according to the method described by Kraus et al. and Ernstbrunner et al. [40, 41]. We will further evaluate the restoration of glenoid cavity depth according to the methodology proposed by Moroder et al. [42, 43]. Screw angle (alpha angle) will be measured for both superior and inferior screws according to the method described by Casabianca et al. [44]. Graft osteolysis will be graded according to Zhu [45] and assessed quantitatively as proposed by Kraus et al. [40], and ROM will be determined as well using a Vicon motion capture system (Vicon, Oxford Metrics, Oxford, UK) consisting of six-camera sampling at 120 Hz already available in our facility. Recording of measurements for this study will be performed by an independent physiotherapist (AS) blinded to the patient allocation group. Return to sport and work absenteeism will be recorded in days and tracked at every follow-up visit.

All complications (including recurrent shoulder dislocation, hematoma, and infection will also be collected) and the aforementioned subjective and clinical scores will be recorded before surgery and at 1.5, 6, and 12 months postoperative.

Following clinical parameters will be collected at the beginning of the study: age, gender, working compensation status (office/mild load/full load), sports activity soliciting shoulders: none, light (< = 4 h/week), intensive (> 4 h/week), hand dominance.

### Participant timeline {13}

**Table:** 

TIMEPOINT**	STUDY PERIOD					
	Enrolment	Allocation	Post-allocation			Close-out
	* − t*_*1*_	0	*3w*	*6w*	*6 m*	*12 m*
ENROLMENT:						
Eligibility screen	X					
Informed consent	X					
Allocation		Surgical stabilization				
INTERVENTIONS:						
*No sling group*						
*Sling group*			X			
ASSESSMENTS:						
*Rowe score*	X			X	X	X
*pVAS, SANE instability*	X			X	X	X
*Satisfaction*						X
*X-ray*	X			X		
*CT scan*	X				X	

#### Sample size {14}

Sample size calculation was based on primary study outcome measurement. Rowe’s score minimal clinically important difference is 9.7 points [46]. The Rowe standard deviation is expected to be around 13.2 points [46]. With a statistical power of 90%, the sample size required to determine whether differences are significant (alpha = 5%) between the control and the experimental group is 40 patients per group. Considering a drop-out rate of 5%, our final sample size is 86 patients, 43 per group.

### Recruitment {15}

Recruitment will be performed by the senior author and principal investigator who operates around 100 shoulder instability cases per year, two-thirds being stabilization according to Latarjet. The recruitment period should thus spread over a 12-month period. Patients are approached by the principal investigator during appointment at his clinic. The principal investigator is further the only shoulder surgery consultant at his institution and is thus able to screen all patients presenting with shoulder instability.

#### Assignment of interventions: allocation

### Sequence generation {16a}

After informed consent is obtained and just past baseline visit, patient will be randomized into a sling and no sling groups. To allocate patients into the two groups, the investigators will use a computer-generated list of random numbers with an allocation of 1:1 using block sizes of four or six using R (version 3.6.2, R Foundation for Statistical Computing, Vienna, Austria). No stratification techniques will be used in the randomization process.

### Concealment mechanism {16b}

An independent researcher from the clinical research department (who is not participating in this study) will keep the randomization list on a secure server that is not accessible to the principal investigator. The allocation will be concealed until the principal investigator (AL) decides to enrol a patient based on defined inclusion and exclusion criteria.

### Implementation {16c}

The principal investigator (AL) will first verify the eligibility of each patient based on study inclusion and exclusion criteria. If the eligibility is confirmed, the principal investigator (AL) will enter the patient baseline information in the CRF. Then, an independent researcher from the clinical research department will inform the principal investigator (AL) of the allocation group, which will be thereafter communicated to the patient.

#### Assignment of interventions: blinding

### Who will be blinded {17a}

The independent physiotherapist (AS) will be responsible for range of motion assessment and will be blinded to the patient allocation group. Radiologic assessment and statistical analysis will be also blinded.

### Procedure for unblinding if needed {17b}

As the intervention is not blinded to the primary investigator and patient, there is no need for an unblinding procedure.

#### Data collection and management

### Plans for assessment and collection of outcomes {18a}

Patients will be screened in the preoperative consultations. Anamnesis, physical examination, and a magnetic resonance arthrography are needed for screening. The randomization procedure will occur on the same day of the intervention after the patient gives written informed consent. Investigators will access the next patient of randomization from the data management system. Patient withdrawal will occur when they withdraw their informed consent, in case of loss of follow-up, or if they do not follow the protocol.

### Plans to promote participant retention and complete follow-up {18b}

The protocol is part of a normal and usual 6-month follow-up for such surgical procedure and therefore no particular plan was developed to maximize retention. Especially, there was no payment to incentivize participants to complete the study.

### Data management {19}

All data will be saved in the corresponding electronic case report form (CRF) and stored on servers (FollowHealth, https://www.follow.fr, 35,000 Rennes, France). Radiologic imaging will be held in La Tour Hospital computer system by the senior surgeon (AL) (32-bit Osirix Version 5.8, Pixmeo SARL, Bernex, Switzerland). The randomization list will be stored on La Tour hospital computer system.

### Confidentiality {27}

A sequential unique patient number (UPN) will be attributed to each patient randomized into the trial. Identification of patients will be stored on a randomization list. Patients must be informed of and agree to data and material handling in accordance with Swiss data protection law.

### Plans for collection, laboratory evaluation, and storage of biological specimens for genetic or molecular analysis in this trial/future use {33}

Given that no biological specimens will be collected in this trial, no specific plan is needed.

#### Statistical methods

### Statistical methods for primary and secondary outcomes {20a}

Statistical analysis will be performed with the “intention to treat” method. Clinical parameters of interest listed above will be compared between the two groups with adapted statistic tests (chi-squared, Wilcoxon, or *T* test), as well as baseline clinical scores. No statistical adjustments on potential confounders are planned, except for predictive analysis (in secondary outcomes). A superiority analysis will be performed with the Student or Wilcoxon test (depending on the distribution of the data). A *p* value of < 0.05 will be considered significant. Clinical parameters of interest (ROWE, VAS, SANE, and ROM) will be compared between groups at 1.5 and 6 months of follow-up. We will also compare the rate of recurrent dislocation, return to sport, work absenteeism, and other complication rates (especially frozen shoulder). When appropriate, secondary outcomes will be compared between groups with superiority analysis, using chi-squared test or Student’s test. Adverse events will be reported and described with percentage and, if adapted, with proportion confidence intervals. In case of missing clinical parameters of interest for primary outcome calculation, patients will be excluded from the study.

### Interim analyses {21b}

No interim analysis has been planned because mobilization and immobilization management are now considered good clinical practices. Therefore, their potential benefits are limited. Moreover, interim analysis would be a waste of statistical power.

### Methods for additional analyses (e.g., subgroup analyses) {20b}

No additional analysis has been planned.

### Methods in analysis to handle protocol non-adherence and any statistical methods to handle missing data {20c}

The analyses were performed following the intention to treat analysis method, known to avoid any bias in superiority trials. The missing data was completed using either the last observation carried forward when possible or by a multiple imputation by chained equation (MICE) method.

### Plans to give access to the full protocol, participant-level data, and statistical code {31c}

The full trial protocol will be made available on the free and participative wiki of BeeMed (https://wiki.beemed.com/view/Anteroinferior_Glenohumeral_Instability, BeeMed, Lausanne, Switzerland) along relevant study results. Anonymized participant-level dataset and statistical code for generating the results will be available upon reasonable request from the corresponding author and principal investigator.

#### Oversight and monitoring

### Composition of the coordinating center and trial steering committee {5d}

The trial is coordinated by a scientific secretary (Anne-Sophie Guillarme) and the clinical research manager of La Tour Hospital (HB) a research scientist under supervision of the senior author and principal investigator (AL). The coordination center meets at least once per month. The steering committee includes an independent chairperson, one shoulder expert and one clinical trials methodologist. The trial steering committee monitors and supervises the progress of the study and meets on regular intervals (quarterly at least).

### Composition of the data monitoring committee, its role and reporting structure {21a}

The abovementioned data monitoring committee is composed of two persons working in a clinical research organization. The role of this monitoring committee is to ensure that the data obtained throughout the study period corresponds to what has been previously defined in the research protocol. Furthermore, this committee will ensure that any adverse event is monitored and reported to the local ethical committee if needed.

### Adverse event reporting and harms {22}

No specific adverse event is expected in the no sling and sling group other than those related to the surgical procedure for which both groups receive standard of care and postoperative visits. Intervention is therefore regarded as low risk. In the unexpected case of an adverse event occurrence, patients will call the investigators, and additional visits will be organized.

A serious adverse event (SAE) is classified as any untoward medical occurrence that:

- Results in death,
- Is life-threatening,
- Requires in-patient hospitalization or prolongation of existing hospitalization,
- Results in persistent or significant disability/incapacity, or.
- Is a congenital anomaly/birth defect.

In addition, important medical events that may not be immediately life-threatening or result in death, or require hospitalization, but may jeopardize the patient or may require intervention to prevent one of the other outcomes listed above should also usually be considered serious.

SAEs should be followed until resolution or stabilization. Participants with ongoing SAEs at study termination (including safety visit) will be further followed up until recovery or until stabilization of the disease after termination.

All SAEs must be reported immediately and within a maximum of 24 h to the Sponsor-Investigator (AL) of the study. The Sponsor-Investigator will re-evaluate the SAE and return the form to the site. SAEs resulting in death are reported to the local Ethics Committee (via local Investigator) within 7 days. The other in the trial involved Ethics Committees who receive SAEs resulting in death in Switzerland via Sponsor-Investigator within 7 days.

### Frequency and plans for auditing trial conduct {23}

The protocol was approved by the local ethical committee board (CCER 2019–02,469) in April 2020. The recruitment started in January 2022. The local ethical committee board (CCER, Commission Cantonale d'Ethique de la Recherche sur l'être humain du canton de Genève) is in charge of auditing the trial conduct.

### Plans for communicating important protocol amendments to relevant parties (e.g., trial participants, ethical committees) {25}

Substantial amendments are only implemented after approval of the CEC and CA respectively.

Under emergency circumstances, deviations from the protocol to protect the rights, safety, and well-being of human subjects may proceed without prior approval of the sponsor and the CEC/CA. Such deviations shall be documented and reported to the sponsor and the CEC/CA as soon as possible.

All non-substantial amendments are communicated to the CA as soon as possible if applicable and to the CEC within the Annual Safety Report. In case substantial amendments are implemented and approved by CEC and CA, the trial protocol will be updated on ClinicalTrials.gov (Identifier: NCT04479397).

### Dissemination plans {31a}

Trial results will be sent to participants and an article submitted to a peer-review journal indexed in PubMed. Moreover, the study will be summarized, and relevant content added on the free and participative wiki of BeeMed (https://wiki.beemed.com/view/Anteroinferior_Glenohumeral_Instability, BeeMed, Lausanne, Switzerland).

## Discussion

This randomized control study is designed to evaluate the impact of sling immobilization on early rehabilitation after open Latarjet surgery and its impact on patient functional outcomes. Rehabilitation after open Latarjet surgery currently remains a matter of surgeon preference rather than driven by a scientific rationale. Rehabilitation is therefore subject to high variability between centers [47]. Only a single study reported short-term outcomes of immediate self-rehabilitation after open Latarjet surgery [24]. According to Roulet et al., the use of self-rehabilitation allowed patients to regain preoperative range of motion after only 3 months, while they found no increase in adverse events, including postoperative hematomas, coracoid graft bony union, and recurrent subluxation or dislocations. On the contrary, patients who did not adhere to immediate postoperative self-rehabilitation during the first postoperative month were found to have significantly more pain as well as limited active forward elevation and internal rotation at 3 months follow-up. As wearing a sling is associated with a higher reported risk of fall, it also seems suitable to avoid unnecessary immobilization [48]. Our results will therefore help to determine if sling immobilization is appropriate after open Latarjet surgery.

Along with functional scores and recurrence rates, return to sport is an essential factor in evaluating the success rate of anterior shoulder stabilization [49]. A fast return to activity and sport seems most suitable. At the same time, the goal of a postoperative sling is to balance the risk inherent to excessive traction onto the coracoid process and stiffness secondary to immobilization, preventing timely return to play in athletes [47, 50]. To our knowledge, all current rehabilitation protocols rely on a sling immobilization [47]. A recent systematic review evaluated an 83.6% rate of return to sport at a mean time of 5 months [49]. Therefore, our study will also give important information on the potential impact of sling immobilization regarding the timing to return to sport.

Finally, one of the most feared complications after open Latarjet surgery is the failure of screw fixation and non-union of the graft. Previous systematic reviews reported early graft complications, including fractures and non-union rates around 1.9–3.2% as the most common complication [51, 52]. However, this rate might be underestimated as most studies relied on standard radiography to assess bony union, with computed tomography studies reporting 7–11.9% non-union rates after open Latarjet [53, 54]. Screws are the most common fixation method of Latarjet. However, results will not allow comparison with other graft fixation techniques, including endobutton fixation, given that initial fixation strength might differ [55, 56].

Another interesting outcome will result from using a motion capture system that allows a precise and reliable measurement of patient range of motion [57]. These findings confirm the benefit of the Latarjet to recovering full range of motion, especially in overhead throwing athletes, especially when facing subcritical glenoid and humeral bone loss [58, 59].

Lastly, a potential benefit of avoiding a sling postoperatively is the prevention of muscle atrophy. Recent studies suggested a negative impact on internal rotation at 90° of abduction after the Latarjet procedure secondary to subscapularis rerouting compared to iliac bone grafts (42, 60).

As aforementioned, the strength of this randomized control trial is the use of computed tomography to confirm bony union after open Latarjet procedure, along with the help of motion capture technology to assess patients’ range of motion. Randomization will ensure certain homogeneity between both groups, which will be operated by the same senior surgeon using the same operative technique.

The main limitation concerns the inherent variability of the patient’s implication towards the self-rehabilitation protocol as well as coping with postoperative sling immobilization after the randomization process. Indeed, patient implication can differ, while randomization and systematic patient education at all follow-up visits should limit their impact on our results.

We are confident that our study will allow precious insights into rehabilitation after open Latarjet surgery.

## Trial status

The trial is currently ongoing. The protocol was approved by the ethical committee board (CCER 2019–02,469) in April 2020 and by ClinicalTrials.gov (Identifier: NCT04479397) in July 2020. The recruitment started in January 2022 and is planned to be completed in January 2023.

## Abbreviations

CA: Competent authority
CEC: Competent Ethics Committee
CCER: Commission cantonale d'éthique de la recherche
CRF: Case report form
CT: Computed tomography
SANE: Single assessment numeric evaluation
UPN: Unique patient number
VAS: Visual Analog Scale A sequential

## Declarations

### Availability of data and materials {29}

All data will be available on request at the following email address: alexandre.laedermann@gmail.com.
